# The Doctor's Critic Examined

**Published:** 1889-07-27

**Authors:** T. G. Headley


### Editor's Letter-Box.

Our Correspondents are reminded that prolixity is a great bar to publication, and that brevity of style and conciseness of statement greatly facilitate early insertion.]

### THE DOCTOR'S CRITIC EXAMINED.

By the Rev. T. G. Headley.

What is nature when we are told to copy it? What is excess when we are told to avoid it? What is reason when in one and the same breadth we are told to use it and to abnegate its use? What is a revelation whilst it is a mystery or unrevealed?

These questions are raised by Miss A. M. F. Cole's criticisms, in your issue of the 13th inst., on the letter headed "The Doctor's Pulpit," which was published in *The Hospital* of the 29th June; and, as I have no doubt whatever that both the Doctor and Miss Cole are most excellent persons, it seems a duty to study the religious difficulties or problems that appear to separate them, in order to reconcile, if possible, propositions that seem momentarily irreconcileable to one or both parties.

First this: What ought we to understand by Nature, when we are told it is something which we ought to copy, follow, or obey in our religion?

Is not our giving obedience to nature equivalent to our obeying a duty and religion? For when a man either as a father or husband, as a brother or friend, flagrantly omits to do some great act of duty, or commits some great offence against his family or society, is he not held up to contempt and scorn as having acted unnaturally and irreligiously? And, therefore, to do our duty is to act naturally and religiously, *i.e.*, according to the teaching or lessons of nature, which (whether revealed miraculously or by evolution) are so innumerable that a life itself is insufficient to exhaust a knowledge of them; *e.g.*, the love of the parent for its offspring is universal and therefore natural, and consequently it is most unnatural when it is wanting in ourselves; for so great is the love of all animals for their offspring that they will defend them when in danger at the risk of their own life. So that when we perform acts of self-sacrifice to save or bless our children, friend or neighbour, we are obeying the law or spirit of our nature as a religious duty, and not committing an excess.

II. What then is an excess? An excess is said to be anything done in extreme, and the poet tells us to avoid extremes, and shun the faults of those who say or do too little or too much; so that if we are ever in danger of doing too little we are also in danger of doing too much. But when Miss Cole would draw a hard-and-fast line between "self-preservation and self-sacrifice," just as though these were antagonistic to one another, and that the law or spirit of Nature and the teaching of Christ were opposed to one another, the distinction is more imaginary than real, because without the preservation of that which alone makes life dear and desirable—*i.e.*, the honour and respect of our neighbours and our own self-esteem—life itself is not worth possessing. And therfore self-preservation oftentimes requires self-sacrifice as the only condition of preserving the respect of our neighbours and our own self-respect; as what man could see his wife, child, or friends in danger, either from drowning or anything else, without risking his life to save them, if there was any chance of saving them by risking his own life. And thus self-preservation requireth self-sacrifice when the love of Christ constraineth us. It may readily be conceded, even by Miss Cole, that the multiplication of church goings, fastings, prayers, and mortifications, are only a means to an end, viz., salvation; and that so long as salvation is uncertain, nothing that is done to obtain it can seem to be excessive, although after it is obtained the same works (if continued) would be in excess and become works of supererogation and efficacious (according to the teaching of the Roman Church) for the salvation of others. But this is not the religion of Christ in the Bible as I understand it. For whilst Ecclesiasticism would have us go out of the world to save ourselves, Christ's teaching would have us go into the world as God's children to save others, because in Christ's great love for us we have seen a manifestation of God's love for us, which must necessarily be salvation. And it seems altogether a mistake of Miss Cole to associate the name of "Gordon" with ritualistic practices, as General Gordon was a man of action, with whom to work for Christ was to pray.

III. What is reason, whilst it is urged that it is more reasonable to obey what is revealed, because of its being beyond reason, than to reason upon it.

But this proposition assumes (*a*) that there is a general agreement as to what has been revealed, and (*b*) that it has already been proved to be beyond reason, and then (*c*) Miss Cole asserts that Faith never contradicts reason. Yet, *if* the faith to which Miss Cole refers is one and the same thing with what she speaks of as having been revealed and being beyond reason, then by what light is Miss Cole able to assert it is not contrary to reason.

Moreover, those who thus dogmatise about "the Faith" and revelation as being beyond reason seem to forget that it is Atheism to speak of something as having been revealed and yet remaining unrevealed and beyond reason, because, even when we assume God to be the author of what is called the Faith or revelation, it only makes the author a God of the dead and not of the living, whilst it remains beyond our reason to fathom and explain. And whilst what has been revealed continues to remain beyond our reason we must be blind, and whilst blind how can the blind see clearly to cast out the mote from their neighbour's eye. And surely it is an abuse of Christ's name to use it as an authority for a multiplication of churchgoings, prayers, fastings, and mortifications, which may be justly called an "an excess of religion," when the one thing alone for which Jesus Christ lived and died was to persuade us to love one another as He loved us, so that peace and goodwill might reign upon earth. But I do not for one moment doubt that Miss Cole is doing her utmost to accomplish this end, and I only ask her to remember Christ tells us that many who are rejected of men are accepted of the Lord, so that the Doctor, whom Miss Cole does not allow to be a Christian, may also be accepted as a good Christian, which I believe him to be, though he may not utter the modern Shibboleth.

